# Protocol for evaluating neuronal activity and neurotransmitter release following amyloid-beta oligomer injections into the rat hippocampus

**DOI:** 10.1016/j.xpro.2025.103712

**Published:** 2025-03-24

**Authors:** Vincent Hervé, Laurie Bonenfant, Mathilde Amyot, Rime Balafrej, Obai Bin Ka’B Ali, Habib Benali, Jonathan Brouillette

**Affiliations:** 1 Department of Pharmacology and Physiology, Université de Montréal, Montréal, QC, Canada; 2 Department of Electrical and Computer Engineering, Concordia University, Montréal, QC, Canada

**Keywords:** Neuroscience, Mass spectrometry, Systems biology

## Abstract

In Alzheimer’s disease, there is an imbalance in neurotransmitter release and altered neuronal activation. Here, we present a protocol approach to analyze neuronal activity by combining local field potential (LFP) recording with microdialysis within the same animal. We describe steps for measuring glutamate and GABA levels following hippocampal amyloid-beta oligomer (Aβo) injections in rats. We then detail procedures for assembling the electrode and cannula, surgical implantation and simultaneous *in vivo* LFP recording, interstitial fluid collection, and Aβo injections.

## Highlights

• Protocol for analyzing neuronal hyperactivity and neurotransmitters in the same animal
• Instructions for the preparation of amyloid-beta oligomers (1–42)
• Guidance on the assembly of the cannula and electrode
• Procedures for surgical implantation of the assembly complex

## Abstract (editor highlights)

Publisher’s note: Undertaking any experimental protocol requires adherence to local institutional guidelines for laboratory safety and ethics.

## Graphical abstract

**Figure:**
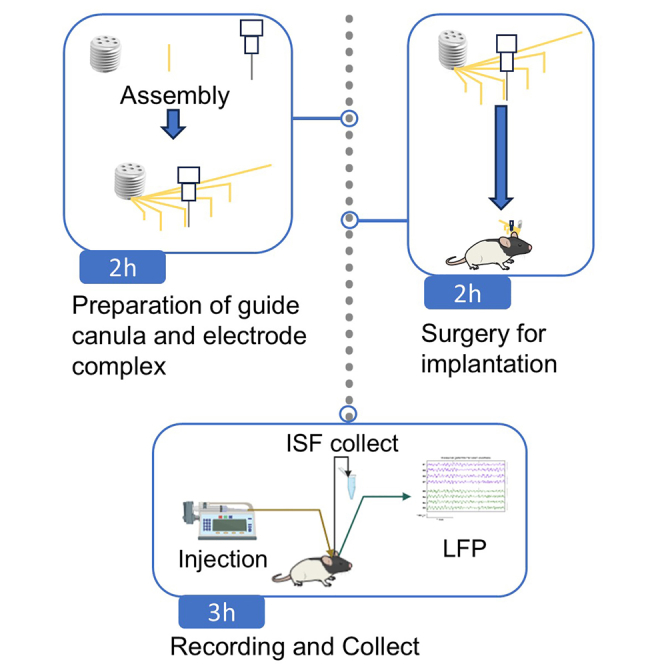

## Before you begin

One of the main neuropathological hallmarks of Alzheimer’s disease (AD) is the accumulation of neurotoxic amyloid-beta oligomers (Aβo), which begins in the brain approximately 15 years prior to the onset of tau pathology, brain atrophy, memory decline, and the clinical diagnosis of AD.^1^^,^^2^ Thus, by acting on Aβo-induced neurodegeneration we could develop therapies that prevent, or at least slow down, the disease as early as possible before neurodegeneration produces irreversible brain damage and severe cognitive deficits.

Based on animal models, *in vitro* experiments, and human studies, neuronal hyperactivity induced by Aβo has emerged as an early functional characteristic of AD, leading to synaptic deficits, memory dysfunction, and neurodegeneration.^3^ Neuronal hyperactivity is a key marker of AD that has been observed in various animal models of the disease as well as in humans.^3^ The extracellular increase in endogenous Aβo, induced in part by the inhibition of its degradation, also enhances the release of glutamatergic vesicles and causes neuronal hyperexcitability in primary cultures and rat hippocampal slices.^4^ In cultures of neurons derived from induced pluripotent stem cells with certain familial AD mutations that allow for Aβ overexpression, a transient increase in Ca^2+^ and excessive neuronal excitability have been observed.^5^^,^^6^

Various studies have elucidated some of the cellular and molecular mechanisms explaining how Aβo can induce neuronal hyperactivity. Indeed, it was suggested that Aβo disrupt the excitation/inhibition balance by reducing inhibitory activity within the GABAergic system, leading to excessive activation of the glutamatergic system in AD mouse models.^7^^,^^8^^,^^9^ Other studies also suggest that the hyperactivity caused by Aβo may be attributed to excessive accumulation of glutamate in the synaptic cleft due to decreased glutamate reuptake by glial and neuronal cells.^10^^,^^11^ However, to date, no study has demonstrated *in vivo* if neuronal activity directly associated with Aβo-induced release of glutamate and GABA during the progression of amyloid pathology. Additionally, it remains unclear whether these phenomena are sufficient to lead to excitotoxicity and neuronal death as observed in the hippocampus during the early stages of AD.

The protocol below outlines the various steps required to study neuronal activity and neurotransmitter quantification in the same animal following injections of Aβo. It describes the procedures for setting up the microdialysis system, performing injections, and recording Local Field Potential (LFP) signals, as well as the surgical steps for electrode and cannula implantation. Finally, it details the recording of neuronal signals and the collection of neurotransmitters during Aβo injections. This novel approach is of interest not only to decipher the molecular and cellular mechanism underpinning soluble Aβo, but any other molecules that might have an impact on neurotransmitter release and neuronal activity in different brain regions of animal models.

Before starting this protocol, it is necessary to order the animals, allow them a period of habituation to their housing environment, and accustom them to handling. Additionally, it is necessary to prepare the Aβo solution in advance.

All procedures with animals were conducted according to the guidelines of the Canadian Council on Animal Care, and the protocol was approved by the *Comité de déontologie de l’expérimentation sur les animaux* of the University of Montreal. Laboratories intending to use this protocol must first obtain approval from their respective institutions.

### Institutional permissions

Experiments on live vertebrates must be conducted in accordance with national guidelines and regulations. Additionally, depending on the legislation of certain countries, verify whether the use of anesthetics requires government authorization. The use of Aβ must also be done after authorization from the health and biosafety committee of the work institution.

### Animal habituation

**Timing: 5 days**

Rats (Males – Long Evans) arrive at the animal facility at 8 weeks of age. The week following their arrival, the animals gradually become accustomed to the experimenters and to being held in restraint. On the first two days, gloved hands are introduced into the cage. On the third day, the animal is placed on a towel held by the experimenter to familiarize it with the scent. From the fourth day until the fifth day, the animal is restrained to simulate an intraperitoneal injection and to mimic the placement of the microdialysis probe.

### Amyloid-beta oligomer preparation

**Timing: 3 h**

This step describes the preparation of amyloid-beta oligomers up to their freezing.

1. Thaw the bottle of Aβ_1-42_ for 10 min at 20°C then centrifugal at 2500 × g at 22°C.

***Note:*** Use the same protocol for the Aβ scramble (control).

***Note:*** During the Aβ handling experiments, it is required at all times to wear a lab coat, safety glasses, and nitrile gloves in accordance with biosafety regulations.

2. Add 500 μL of 1,1,1,3,3,3,-Hexafluoro-2-propanol (HFP) under the chemical hood.
  a. Vortex during 1 min 30 s by inverting the bottle every 30 s.
  b. Evaporates the HFP while maintaining a light flow of nitrogen in the bottle.
3. Prepare Tris-EDTA buffer (50 mM Tris, 1 mM EDTA) under sterile conditions:
  a. Add to a 50 mL tube: 2.5 mL of 1 M Tris (pH 7.4), 500 μL of 100 mM EDTA, 47 mL sterile water (Baxter JF7624).
  b. Filter this buffer dropwise through a 0.22 μm filter (Sigma Aldrich Z260347-1PAK) using a 20 mL syringe.
4. Add 500 μL of DMSO in the bottle and vortex.
5. Equilibrate the Hitrap desalting column with 25 mL of Tris-EDTA buffer.
  a. Apply 500 μL of Aβ-DMSO in Hitrap desalting column.
  b. Add 1 mL of Tris-EDTA buffer and collect the last 200 μL in tube named Aβo.
  c. Add another 1 mL of Tris-EDTA buffer.
    i. Collect the first 800 μL in low-adhesion tube named Aβo.
      ***Note:*** This tube contains the highest concentration of Aβ.
    ii. Collect the last 200 μL in a tube named Aβ_2_.
6. Determine the concentration of each tube (Aβ_before_, Aβo, Aβ_after_) with Pierce BCA Protein Assay Kit.
  ***Note:*** Also remember to measure the absorbance of the Tris-EDTA buffer.
  a. Shake the low-adhesion 96-well plate during 10 s at 120 rpm every 15 min during the 45 min incubation.
  b. Prepare 200 aliquots of 4 μL each from the 800 μL Aβo solution using low-adhesion tubes and tips. Immediately place the aliquots at −80°C for storage until use.
  c. Measure absorbance of the plate at 562 nm and the concentration in tube Aβo using the standard line obtained with the absorbances of the different concentrations of BSA measured on the spectrophotometer.
    ***Note:*** If the elution of Aβ across the HiTrap desalting column is performed correctly, Aβ should be concentrated entirely in the Aβo tube, with no presence in the tubes before (Aβ_before_) or after (Aβ_after_) its elution. The concentration in the Aβo tube should be approximately 0.3 μg/μL.
  d. Determine the volume of Tris-EDTA buffer to add to obtain a final concentration of 0.2 μg/μL.

## Key resources table

**Table:** 

REAGENT or RESOURCE	SOURCE	IDENTIFIER
**Chemicals, peptides, and recombinant proteins**		

Aβ1-42	rPeptide	*#A-1163-2*
Alfaxalone (10 mg/mL)	Jurox	424-520425
1,1,1,3,3,3,-Hexafluoro-2-propanol (HFP)	Acros Organics	147541000
EDTA	Fisher	BP2927-100
Tris	Fisher	BP153-1
DMSO	Sigma	D4540
Acrylic liquid	Yates Motloid Company	44119
Dexmedetomidine (0.5 mg/mL)	Orion Pharma	02333929
Meloxicam (5 mg/mL)	Vetoquinol	02487225
Buprénorphine slow release 3 mg/mL	Chiron	RxN800431
Atipamazole	Orion Pharma	02237744
Lidocain	Odan Lab	02083795
Bioderm	Odan Lab	00621366
Artificial cerebrospinal fluid	Harvard Apparatus	59-7316

**Experimental models: Organisms/strains**		

Long Evans rats; outbred, male, 3 months	Charles River	006L/E

**Software and algorithms**		

Harmonie	Natus, Middleton	N/A
EDF browser	https://www.teuniz.net/edfbrowser/	N/A

**Other**		

Microdialysis and infusion probe	Basics	MD-2264
Guide cannula	Basics	MD-2250
6 Channel electrode-Platinum Teflon (uncut)	P1 Technologies	E363/6/SPC
6 Channel electrode pedestal	P1 Technologies	MS363
Filter 22 μm	Sigma-Aldrich	Z260347-1PAK
Hitrap desalting column	Cytiva	17140801
Pierce BCA Protein Assay Kit	Thermo Scientific	23227
PE50	Plastic One	C232CT

## Step-by-step method details

### Preparation of guide cannula and electrode complex

**Timing: 2 h**

This step involves assembling the electrode complex and guide cannula to prepare them for implantation in the animal.

**Figure 1:**
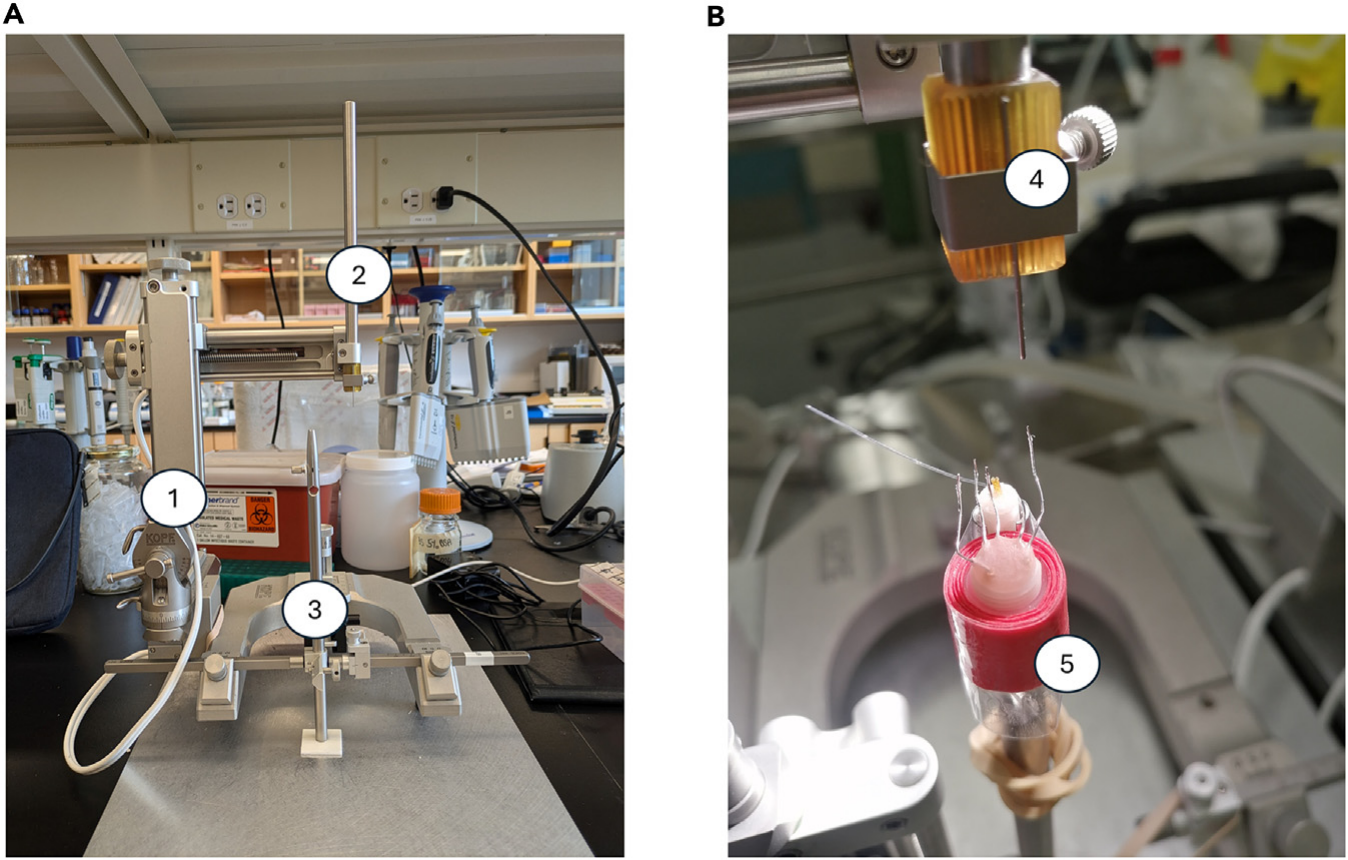
Preparation of guide cannula and electrode complex (A) Setup for assembling the electrodes and guide cannula using a stereotaxic apparatus (1). The standard electrode holder with cut needle (2) and cannula holder (3) are shown. (B) The pedestal containing the five electrodes and the guide cannula (5) is shown, along with the cut needle on the standard electrode holder (4).

***Note:*** If the cap is too large to fit into the cannula holder, it can be sanded down beforehand.

1. Ensure that the stylet of the cap is the same length as the microdialysis probe.
  a. Place the cap in the guide cannula and then into the cannula holder (Figure 1A, item 3; Kopf instruments - Model 1766-AP).

2. Place the cannula holder with the guide cannula perpendicular to the stereotaxic table, with the cannula facing upwards.
3. Place a cut needle at the end of the standard electrode holder (Figure 1A, item 2; Kopf instruments - Model 1770) which is attached directly to the stereotaxic apparatus (Figure 1A, item 1) to adjust the precise coordinates.
4. Place each electrode in the 6-channel electrode pedestal.

**CRITICAL:** Electrodes are very fragile when connected to the pedestal.

5. Place the cap on the pedestal and wrap it with sturdy scotch tape (in red Figure 1 **(5)**) to create an extra thickness of approximately 2 mm.

***Note:*** This step is important so that the animal can be connected without obstruction during LFP recordings.

6. Wrap the pedestal with scotch tape to fix it with the guide cannula (5).
7. Place and adjust all the electrodes using the stereotaxic apparatus according to the tip of the stylet inserted into the guide cannula.

***Note:*** This serves as the “0” reference point for the coordinates (mediolateral (ML), anteroposterior (AP), dorsoventral (DV)) for positioning each electrode.

8. Adjust each electrode according to the following coordinates using the needle of the standard electrode holder attached to the stereotaxic apparatus (Table 1).

**Table 1:** Coordinates of the different regions where the electrodes are implanted, with the guide cannula and its electrode (right hippocampus) as the reference point

(mm)	Right hippocampus (rHp)	Left hippocampus (lHp)	Right RSC	Right TeA	Right prefrontal cortex (Cg1, Cg2)
ML	0,00	−3,20	−0,80	5,44	−1,00
AP	0,00	0,00	2,00	2,20	−5,50
DV	0,00	0,00	1,00	−2,40	0,60
9. Trim the electrodes to match the specified coordinates.
  a. Carefully scrape off less than 1 mm from the tip of each electrode to remove the insulating layer.
  b. Apply a few drops of dental cement to the electrode side of the pedestal to secure the joint between the electrodes and the pedestal.

### Surgery to implant the electrode-cannula complex

**Timing: 2 h**

10. Perform surgeries in a sterile environment using gloves, a mask, and a hair bonnet during the procedure.

***Note:*** All surgical tools are pre-cleaned and sterilized by autoclaving.

***Note:*** At 10 weeks of age, the rats undergo surgery to implant the electrodes and the guide cannula for the microdialysis probe.

**Figure 2:**
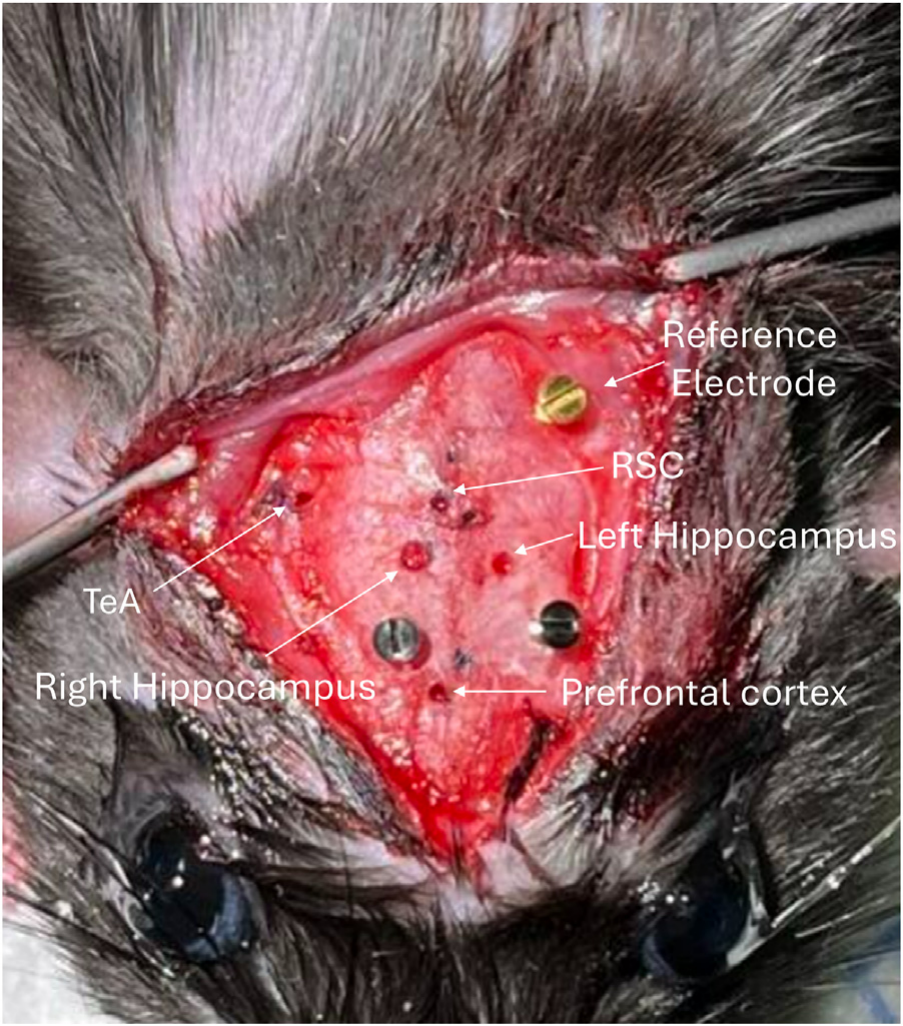
Hole placement for electrode positioning in the rat brain with 3 screws
  c. Replace the standard electrode holder containing the needle with the cannula holder containing the electrode and cannula assembly on the stereotaxic frame. Adjust the arm with the cannula holder so that the electrodes align directly with the drill holes.
  d. Wrap the solder wire around and solder the gold-plated screw (the reference electrode) located above the cerebellum.
    ***Note:*** The cerebellum is an ideal control structure for recording reference signals in LFP measurements.

11. Administer a subcutaneous injection of slow-release buprenorphine (72-h formulation) at least 5 h prior to surgery.
12. Anesthetize the rat with an intraperitoneal injection of a cocktail containing alfaxalone (20 mg/kg) and dexmedetomidine (0.1 mg/kg).
  ***Note:*** Ensure the absence of reflexes before proceeding.
  a. Inject Meloxicam 1 mg/kg subcutaneously.
  b. Shave the animal at the incision site with clippers before placing it on the stereotaxic frame.
  c. Place the animal on the stereotaxic apparatus using ear bars to immobilize the head. The animal is placed on a heating mat maintained at 37°C.
  d. Position the mask over the rat’s nose to maintain anesthesia with isoflurane at a flow rate of 0.5–1.0%.
    **CRITICAL:** After fitting the mask and ear bars, ensure the head remains stable when pressure is applied.
  e. Apply lubricating eye ointment during anesthesia to prevent dryness.
  f. Sterilize the incision area with a swab containing a solution of 2% w/v chlorhexidine gluconate and 70% isopropyl alcohol (3M SoluPrep Swab 102.09) followed by a final soaking with a disinfectant solution (Betadine).
  g. Make an incision in the skin above the skull.
  h. Locate the bregma and lambda, and mark them with a felt-tip pen.
    ***Note:*** Here, the bregma serves as the “0” reference point for the coordinates on the stereotaxic device.
    ***Note:*** To ensure the skull is flat, a difference of 0.05 cm or less in the dorsoventral and mediolateral axes is considered acceptable.
13. Mark each point corresponding to the electrode site as in Table 2.
  a. Drill a hole for each electrode using a 1/32″ drill bit, and use a 3/64″ drill bit for the guide cannula and the adjacent electrode.
    ***Note:*** Additionally, make two more holes with a 1/32″ drill bit to secure the mounting with two screws placed on either side of the bregma.
  b. Add a hole for the gold screw that serves as the reference electrode. The screw is placed above the cerebellum, which is known to be a control zone.
    ***Note:*** The cerebellum is highly vascularized, so if hemorrhaging occurs, wait for it to stop before placing the screw (Figure 2).

**Table 2:** Stereotaxic coordinates of the different brain regions where the skull holes are made for the implantation of our electrode and cannula system

(mm)	Right hippocampus	Left hippocampus	Right RSC	Right TeA	Right prefrontal cortex	Reference electrode
ML	1,60	−1,60	0,80	7,04	0,60	−0,94
AP	3,80	3,80	5,80	6,00	−1,70	10,50
DV	−3,60	−3,60	−2,60	−6,00	−3,00	N/A

RSC = retrosplenial cortex, TeA = temporal association cortex, and PFC = prefrontal cortex.
14. Apply acrylic dental cement around the guide cannula, the electrode pedestal, and the screws to secure the setup.

**Figure 3:**
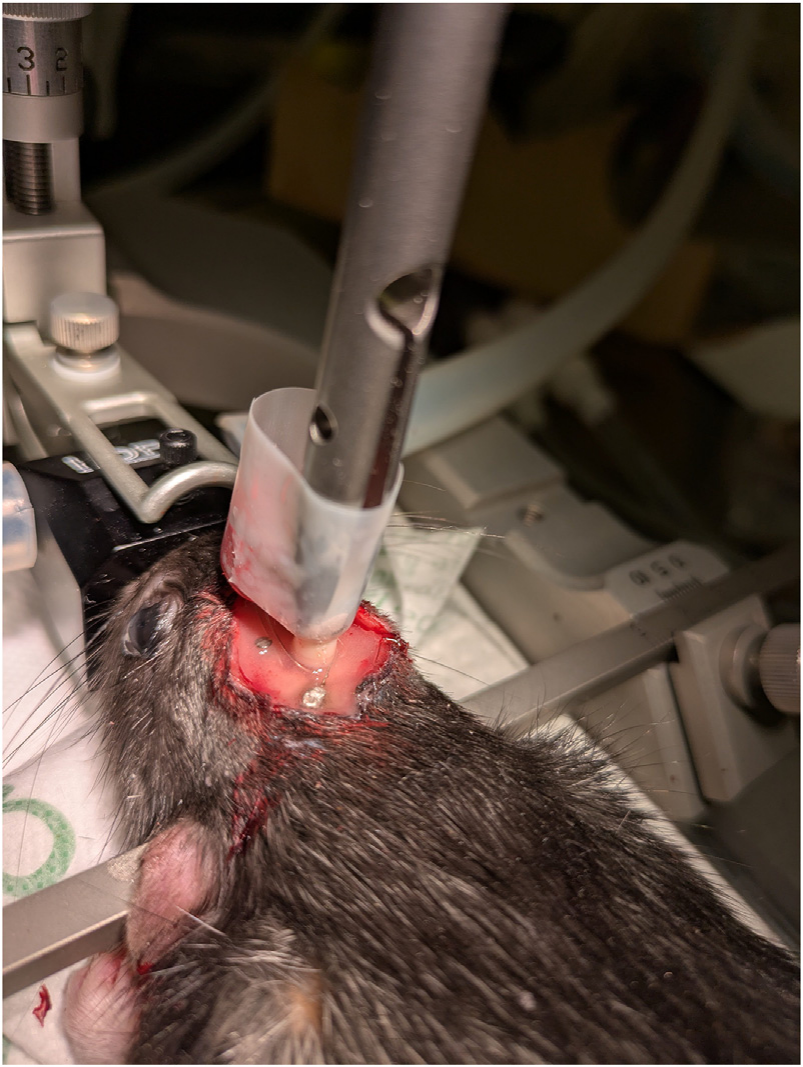
Dental cement to secure the implant on the rat’s skull

***Note:*** Remove the tape binding the pedestal and the guide cannula to improve accessibility and facilitate better fixation of the assembly (Figure 3).

**CRITICAL:** Apply multiple layers of dental cement to ensure the assembly is firmly secured and to prevent any movement.

15. Apply sutures to close the skin using 4-0 surgical thread.
  a. Trim the rat’s nails once the sutures are complete.
  b. Administer 3 mL of saline subcutaneously at 37°C. Apply a cream mixture of lidocaine and polysporin to the incision site daily for 2 days following the surgery.
  c. Inject Atipamezole at an equivalent dose (v/v) of Dexmedetomidine to induce rapid awakening of the animal. Place the rat in an oxygen chamber heated to 37°C.
16. Return the rat to its cage and housing room once it wakes up and can walk.

### Recording and collection of interstitial fluid

**Timing: 3 h**

This step details the entire procedure for Local Field Potential recording sessions and the collection of interstitial fluid (ISF) fractions by microdialysis.

17. Bring the rats into the LFP recording room at 12 weeks of age and 2 weeks after surgery, then allow them a 30-min habituation period.
18. Prepare the setup for microdialysis during this 30-min period:
  a. Take 1 mL of artificial cerebrospinal fluid (aCSF) (Harvard Apparatus) with a 3 mL syringe.
  b. Place the syringe on a pump (CMA pump).
  c. Cut the needle 23G to remove the pointed tip, and connect the syringe to a swivel (Instech 375/D/25) with PE50 tubing.
  d. Connect the swivel to the microdialysis probe (yellow channel).
  e. Connect the microdialysis probe (green channel) to the swivel with microtubing (Basics MD-1511).
  f. Calculate the volume between the microdialysis outlet and the fraction collection to adjust the timing of collection with LFP recording.
19. Set the pump flow rate to 1 μL/min and ensure that the aCSF is circulating properly by observing the appearance of a drop at the end of the circuit.
20. Place the rat in a cage and position the microdialysis probe on the animal. Allow a 1-h equilibration period for the microdialysis probe.
  ***Note:*** During this period, check the volume of the fractions multiple times to ensure 10 μL per 10 min.
  **CRITICAL:** Monitor the animal to prevent it from biting the tubes, which could cause leaks and require repeating the experiment.
  a. Calculate the volume between the **microdialysis** probe outlet and the collection (7 μL), connect the animal for LFP recording 7 min before starting the ISF fraction collection.
    ***Note:*** Keep the animal awake throughout the experiment.
  b. After 1 h of equilibration, collect fractions every 10 min, each with a volume of 10 μL. Add 1 μL of 0.25 mol/L perchloric acid to each fraction to prevent analyte degradation.
  c. After 30 min of microdialysis, perform an injection of Aβo (preparation described in “before you begin”) at a concentration of 0.2 μg/μL using a microsyringe (10 μL) pump at a flow rate of 1 μL/min for 2 min.
    ***Note:*** The injection channel is the red channel in the microdialysis probe.
    ***Note:*** Keep the Aβo solution at 20°C for 1 h before injection to allow the oligomerization of Aβ (Figure 4).

**Figure 4:**
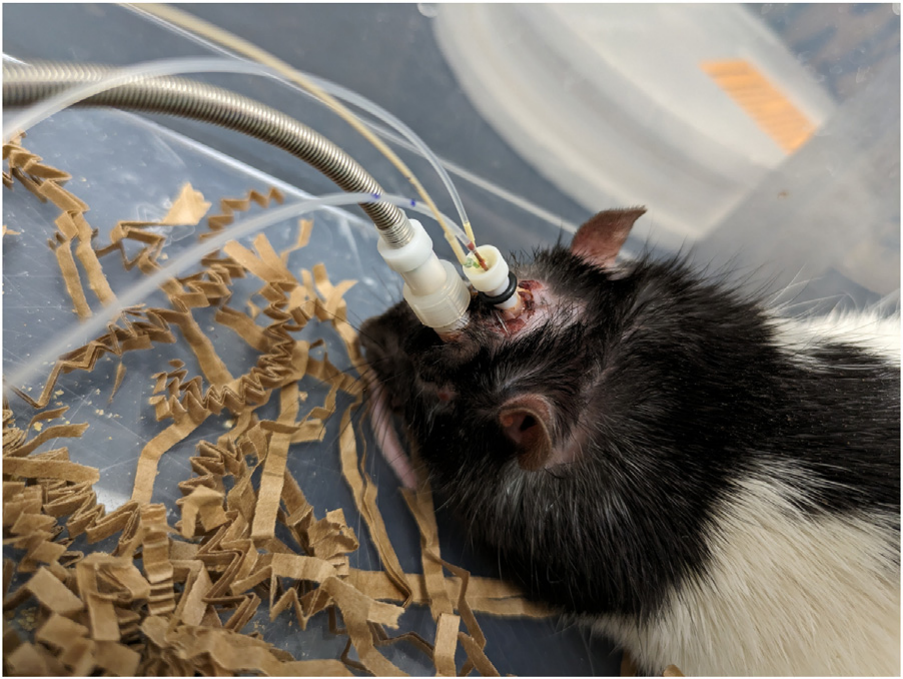
Rat connected for LFP recording, Aβ injection, and microdialysis
  d. Allow 8 min after the injection before removing the injection channel.
    ***Note:*** The LFP signals are amplified using a Lamont amplifier, sampled at 1024 Hz, and filtered with the commercial software Harmonie (Natus, Middleton, WI, USA) as previously described.^12^
21. After the experiment, disconnect the animal from the microdialysis and LFP channels, and return it to its cage.
  a. Clean the probe by flushing the injection and microdialysis channels with water.
  b. Connect the green and yellow channels to PE-50 tubing filled with water, and plug the red channel. Leave probe to soak in water until next use.
    ***Note:*** Store the probe in water within its cap at 4°C.
  c. Clean the swivel with a few microliters of water, then dry it with a connected syringe at a flow rate of 10 μL/min.

## Expected outcomes

**Figure 5:**
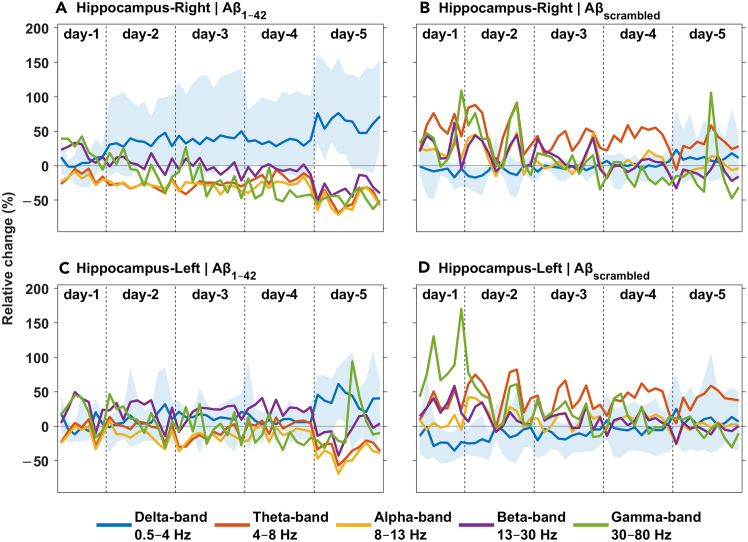
Effects of Aβo injections over 5 days on LFP power Comparisons were made between the right hippocampus (injection site) and the left hippocampus for both the Aβo group (*n* = 4) and the Aβscr control group (*n* = 5). (A) and (B) represent the relative percentage changes in each frequency band in the right hippocampus injected with Aβo and Aβscr, respectively. (C) and (D) represent the relative percentage changes in the left hippocampus injected with Aβo and Aβscr, respectively. A semi-transparent ribbon is overlaid on the delta band to show the minimum and maximum values, calculated across subjects. Significance was assessed at a threshold of *p* < 0.05. Statistical trends were analyzed using linear mixed-effects models for each frequency band, with fixed effects for time, group, and hemisphere, and random intercepts for subject-level variability.

Using the novel animal model described here, we observed that soluble Aβo injections over 5 days in the right hippocampus of rats induced a clear time-dependent increase in delta power (slope = 0.73, SE = 0.13, *p* < 0.001) and a progressive decrease in higher <listaend>frequencies, including theta (slope = −0.52, SE = 0.22, *p* = 0.018), alpha (slope = −0.58, SE = 0.16, *p* < 0.001), and beta (slope = −0.57, SE = 0.21, *p* = 0.006), in the injected hippocampus (Figure 5A). Gamma power also shows a decrease over time (slope = −0.62, SE = 0.37, *p* = 0.094), though this effect approaches marginal significance. This pattern of increased slow-frequency and decreased higher-frequency power reflects the slowing of neuronal oscillations commonly associated with AD pathology. These results are in accordance with what we observed in a previous study,^13^ confirming that Aβo injections consistently lead to these time-dependent changes in power. In the left hippocampus with no injection (Figure 5C), the Aβo group initially showed stable trends, with a late emergence of increased delta power and decreased higher-frequency power on day 5, resembling those observed in panel (a). This suggests a delayed or propagating effect from the right hippocampus. In contrast, the Aβscramble (Aβscr) control group showed no significant changes in power over time, with no substantial deviations from baseline (Figures 5B and 5D).

In future studies, this animal model could allow for the investigation of the impact of hippocampal Aβo injections on other brain regions, particularly the default-mode network (DMN), where functional connectivity disruptions are detected in early AD.^14^ The DMN is involved in several high-level cognitive processes such as semantic memory, social cognition, and decision-making,^15^ and represents a network of brain regions that are typically more active during rest or when attention is not focused on the external environment.^16^

By implanting three additional electrodes in different areas of the DMN (PFC, RSC, TeA), this animal model enables the assessment of whether Aβo accumulation in the hippocampus affects its interactions with this neural network. Specifically, this approach allows for the analysis of frequency band profiles and functional connectivity measures (e.g., coherence, phase-amplitude coupling) between the hippocampus and DMN regions across different time points. This will help determine whether similar network-wide changes occur alongside those observed in the hippocampus during the injection period.

Moreover, we were able to detect and quantify the neurotransmitters GABA and Glutamate in the ISF fractions collected during our recording periods. In Table 3, we present the quantifications before, during, and after Aβ injection for a rat over a period of 5 days. These results demonstrate the ability of our method to analyze these neurotransmitters. The analytical approach is based on a previous study.^17^ In addition to detecting Glutamate and GABA, this method also enables the quantification of serine, glutamine, and taurine, allowing for extensive data acquisition.

**Table 3:** Quantification of GABA and Glutamate during Aβ injection by liquid chromatography coupled with mass spectrometry

Samples	GABA (μg/mL)	Glutamate (μg/mL)
D1-PRE-INJ	0,003	0,857
D1-INJ	0,005	0,910
D1-POST-INJ	0,005	0,900
D2-PRE-INJ	0,005	1,112
D2-INJ	0,007	0,853
D2-POST-INJ	0,005	0,612
D3-PRE-INJ	0,006	1,525
D3-INJ	0,005	0,701
D3-POST-INJ	0,004	0,299
D4-PRE-INJ	0,006	1,041
D4-INJ	0,006	0,837
D4-POST-INJ	0,004	0,533
D5-PRE-INJ	0,005	1,045
D5-INJ	0,005	1,031
D5-POST-INJ	0,003	1,583

This *in vivo* model can also be used to explore whether changes in neuronal dynamics are directly related to glutamate and GABA release levels following Aβo injection into the hippocampus. Coupling LFP data with microdialysis measurements of glutamate and GABA release offers a powerful approach to understanding neuronal dynamics at both functional and biochemical levels. LFP data reflect the summed electrical activity of neuronal populations, capturing oscillatory patterns and synaptic potentials associated with neuronal communication. By simultaneously measuring glutamate and GABA release, we gain direct insight into the excitatory and inhibitory balance underlying this activity. Overall, integrating LFP recordings with microdialysis-derived neurotransmitter measurements bridges the gap between electrical signaling and the underlying neurochemical processes, offering a holistic view of neuronal dynamics in health and disease. For instance, in models of neurological disorders such as epilepsy or Alzheimer’s disease, coupling LFP with neurotransmitter data could elucidate how dysregulated excitatory-inhibitory balance contributes to altered brain activity. Using another group of rats, neurodegeneration, along with astrocytic and microglial activation, can be assessed via immunofluorescence using antibodies targeting NeuN (neuronal marker), GFAP (astrocyte marker), and IBA1 (microglia marker) proteins after different intervals of Aβo and Aβscr injections. This will allow for the evaluation of neuronal death, astrogliosis, and microgliosis in brain regions of interest, potentially linking these molecular and cellular changes to the observed alterations in neuronal activity and connectivity.

## Limitations

Our animal model leverages the microdialysis technique to simultaneously detect multiple neurotransmitters in the same animal and measure absolute neurotransmitter concentrations rather than relative or indirect values. This approach enables to precisely determine whether hippocampal release of neurotransmission-related molecules is directly associated with alterations in LFP power across canonical frequency bands following Aβo injections. Since the effects of Aβo on different frequency bands unfold over hours and days, as shown in Figure 4, the microdialysis fractions collected every 10 min for neurotransmitter quantification via mass spectrometry provide an appropriate level of precision to assess the impact of Aβo on neurotransmitter release when oscillatory patterns are altered. For researchers aiming to more closely correlate neurotransmitter dynamics with LFP activity, an electrochemical probe with a temporal resolution of 1 s could be used instead of a microdialysis probe. However, this method has drawbacks: it can only measure one neurotransmitter at a time, and some neurotransmitters (such as GABA) cannot be detected using this approach. Another alternative is fiber photometry, which allows for an even closer correlation between neurotransmitter activity and LFP signals. However, this technique also has limitations. It does not enable the simultaneous measurement of multiple neurotransmitters, offers limited flexibility in fiber placement, and provides only semi-quantitative data, capturing relative changes in neurotransmitter concentration dynamics rather than the absolute quantifications of glutamate and GABA levels that microdialysis offers.

## Troubleshooting

### Problem 1

The electrode pedestal is detached from the animal after the first day of LFP recording.

### Potential solution

We encountered this issue with our first batch of animals and addressed it by increasing the number of screws on the animal’s skull from one to three. Since then, the problem has not recurred. Additionally, during the surgery, ensure that the pedestal and guide cannula are thoroughly coated with dental cement to secure the assembly. The screws will aid in this fixation. Make sure to wait until the dental cement has fully solidified before proceeding.

### Problem 2

The collection volume is not 10 μL or no fluid is emerging from the tube after microdialysis.

### Potential solution

• Check all the tubing connections to ensure there are no leaks at their junctions, and inspect the wires, as the rat may have bitten one. To prevent this, carefully monitor the animal and use shorter tubing to minimize accessibility and reduce the risk of biting.
• If the microdialysis probe is blocked, restart the experiment with the probe balanced if the injection has not yet occurred. If a tube is chewed, replace the tubing and restart the experiment. Ensure the probe is not blocked in either the microdialysis or injection channels before placing it on the animal.

## Resource availability

### Lead contact

Further information should be directed to and will be fulfilled by the lead contact, Jonathan Brouillette (Jonathan.Brouillette@umontreal.ca).

### Technical contact

Questions about the technical specifics of performing the protocol should be directed to and will be answered by the technical contact, Vincent Hervé (Vincent.Herve@umontreal.ca).

### Materials availability

This protocol did not generate new unique reagents.

### Data and code availability

Upon request, datasets and code are available from the corresponding authors.

## Acknowledgments

The authors are thankful to Ian Massé and Louis De Beaumont for their help and advice in setting up the microdialysis procedure. The authors also want to thank Louiza Mahrouche and Mihaela Friciu from the Platform of Biopharmacy at the University of Montreal for quantifying neurotransmitters by high-performance liquid chromatography coupled with mass spectrometry.

The research has been funded by a grant from the Canadian Institutes of Health Research to J.B., a scholarship from the Fonds de recherche du Québec - Santé to V.H., and a scholarship from the PRogramme d’Excellence en Médecine pour l’Initiation En Recherche to R.B. and L.B. H.B. and O.B.K.A. are funded by a Canada Research Chair from the Natural Sciences and Engineering Research Council of Canada (grant number NC0981).

## Author contributions

Conceptualization and methodology, V.H., L.B., M.A., R.B., and J.B.; investigation, V.H. and J.B.; data analysis, O.B.K.A. and H.B.; writing, V.H. and J.B.; funding acquisition, supervision, and project administration, J.B. All authors approved the final version of the manuscript.

## Declaration of interests

The authors declare no competing interests.
